# Detection of independent associations in a large epidemiologic dataset: a comparison of random forests, boosted regression trees, conventional and penalized logistic regression for identifying independent factors associated with H1N1pdm influenza infections

**DOI:** 10.1186/1471-2288-14-99

**Published:** 2014-08-26

**Authors:** Yohann Mansiaux, Fabrice Carrat

**Affiliations:** 1 INSERM, UMR_S 1136, Institut Pierre Louis d’Epidémiologie et de Santé Publique, F-75013 Paris, France; 2 Sorbonne Universités, UPMC Univ Paris 06, UMR_S 1136, Institut Pierre Louis d’Epidémiologie et de Santé Publique, F-75013 Paris, France; 3 Public Health Unit, Saint-Antoine Hospital, 75012 Paris, France

**Keywords:** Data mining, Random forest, Boosted regression trees, LASSO, Logistic regression, Influenza

## Abstract

**Background:**

Big data is steadily growing in epidemiology. We explored the performances of methods dedicated to big data analysis for detecting independent associations between exposures and a health outcome.

**Methods:**

We searched for associations between 303 covariates and influenza infection in 498 subjects (14% infected) sampled from a dedicated cohort. Independent associations were detected using two data mining methods, the Random Forests (RF) and the Boosted Regression Trees (BRT); the conventional logistic regression framework (Univariate Followed by Multivariate Logistic Regression - UFMLR) and the Least Absolute Shrinkage and Selection Operator (LASSO) with penalty in multivariate logistic regression to achieve a sparse selection of covariates. We developed permutations tests to assess the statistical significance of associations. We simulated 500 similar sized datasets to estimate the True (TPR) and False (FPR) Positive Rates associated with these methods.

**Results:**

Between 3 and 24 covariates (1%-8%) were identified as associated with influenza infection depending on the method. The pre-seasonal haemagglutination inhibition antibody titer was the unique covariate selected with all methods while 266 (87%) covariates were not selected by any method. At 5% nominal significance level, the TPR were 85% with RF, 80% with BRT, 26% to 49% with UFMLR, 71% to 78% with LASSO. Conversely, the FPR were 4% with RF and BRT, 9% to 2% with UFMLR, and 9% to 4% with LASSO.

**Conclusions:**

Data mining methods and LASSO should be considered as valuable methods to detect independent associations in large epidemiologic datasets.

## Background

“Big data” [1] in information science refers to the collection and management of large and complex datasets. Big data is steadily growing in biomedicine with the development of electronic medical records, increased use of high-throughput technologies, and facilitated access to large environmental database [2-6]. In epidemiology, the collection of hundreds to thousands of covariates is common in large-scale cohort studies and offers new challenges for the discovery of associations between individual or collective exposures and a health outcome. The use of specific methods to explore these associations, without any pre-specified hypothesis, therefore becomes essential.

In hypothesis-driven epidemiology, the search for associations involves statistical modeling and testing of the relationships between one or several covariates and the outcome. Logistic regression is the most widely used model when the outcome follows a binomial distribution. The usual epidemiologic analytic framework consists in testing the association between each covariate and the outcome through univariate logistic models; a subset of those covariates is then selected for multivariate logistic models based on some quantile of the test statistic for the covariate coefficient under the null hypothesis, *i.e.* the Pvalue. This framework is the reference method in epidemiology for variable selection, and the use of alternative approaches remains uncommon [7,8]. With large datasets, the number of covariates selected in the univariate analyses can be high. As multivariate logistic regression can handle a limited number of covariates simultaneously [9], it might therefore be poorly adapted to large epidemiologic datasets for identifying independent associations.

“Data mining”, a term which appeared in the early 1990’s [10], describes data-driven analysis without any *a priori* hypothesis about the structure or the potential relationships that could exist in the data. Data mining applications are broad, ranging from consumption analysis to fraud detection in high-dimensional databases [11]. Data mining methods are non-parametric, more flexible than statistical regression methods, and are able to deal with a large number of covariates. Several studies have compared the performances of logistic regression and data mining methods for predicting a health outcome without clear conclusions about the superiority of one of these methods over the others [12-17]. Most studies explored classification and regression trees, artificial neural networks or linear discriminant analysis, but only a few focused on more recently developed “ensemble-based” methods such as random forests or boosted regression trees [13,16,17].

Shrinkage methods, such as the Least Absolute Shrinkage and Selection Operator (LASSO) [18], have been developed to overcome the limitation of usual regression models when the number of covariates is high. However, LASSO logistic regression remains unfamiliar to epidemiologists and few applications of this method have been found [19,20].

We hereby performed a comparison of two data mining methods, random forests and boosted regression trees, with the conventional multivariate logistic regression and with the LASSO logistic regression for identifying independent associations in a large epidemiologic dataset including hundreds of covariates. Random forests and boosted regression trees were chosen among data mining methods for their ability to provide quantitative information about the strength of association between covariates and the outcome. The methods were used to detect covariates associated with H1N1 pandemic (pdm) influenza infections. We also assessed the performance of these methods to detect associations through simulations.

## Methods

### Data source

We used data from the CoPanFlu France cohort whose aim was to study the risk of influenza infection. Briefly, the cohort includes 601 households randomly selected between December 2009 and July 2010 and followed using an active surveillance system in order to detect influenza-like illness symptoms over two consecutive influenza seasons (2010–2011 and 2011–2012). More details about the study protocol, data collection and representativeness of households can be found elsewhere [21]. Ethics approval was given for this study by the institutional review board “Comité de Protection des Personnes Ile-de-France 1” and written informed consent was obtained for all participants.

The outcome of our study was H1N1pdm influenza infection during 2010–2011 season, defined as either a positive H1N1pdm RT-PCR [22] or a positive H1N1pdm RespiFinder assay [23] on a nasal swab collected during winter 2010–2011 or a seroconversion (4-fold increase of Haemagglutination inhibition (HAI) antibody titer [24] between post and pre-seasonal serum samples). Infection status for the 2010–2011 season was available in household members from 498 households. To neutralize within household correlation and ensure statistical independence of individuals included in the analysis, we adopted a case–control selection strategy. One case was sampled from each household where at least one influenza infection was detected and one control was selected from each household where no influenza infection occurred. Our analysis therefore focused on 498 subjects, 68 (14%) cases and 430 controls. Association with the outcome was explored for 303 covariates (a complete description can be found in Lapidus *et al.*[25]).

The mean age of study subjects was 44.3 years (SD = 21.1); 42% (208 individuals) were male. A total of 215 subjects (43%) had at least one history of chronic disease. The proportions of seasonal and pandemic vaccines recipients for 2009–2010 season were 19% and 10%, respectively. The mean number of subjects per household was 2.5 (SD = 1.3) and the number of children per household was 0.5 (SD = 0.9).

### Methods for detection of independent associations

#### Random forests (RF)

Random Forests models were proposed by Leo Breiman [26]. RF consists of an ensemble of classification and regression trees. Each tree of the random forest is built as followed: a bootstrap sample of the original dataset is drawn with replacement. The rest of the observations compose the “out-of-bag” sample, used to assess the performances of the selected tree. At each node of the tree, a random subset of covariates is selected (usually as much as the square root of the total number of covariates). Selection of a covariate to split a “parent” node into two “child” nodes is the covariate among the subset leading to the largest decrease in the Gini impurity criterion, that is, for a binary outcome, 1 – p^2^ – (1 – p)^2^ with *p* the proportion of individuals classified with the outcome (the influenza infection in our case). The partitioning process is iterated until the final nodes contain only individuals belonging to the same classes or until they contain only one individual. The tree is then used to classify every individual in the “out-of-bag” sample. This process is repeated until a pre-specified number of trees is reached (one thousand). Note that prediction for each individual is based on the averaged predictions over all trees. To rank potential relevant associations with covariates selected in the RF, we used the importance score, *i.e.*, the decrease in Gini impurity criterion from splitting on the covariate, averaged over all trees.

#### Boosted regression trees (BRT)

Boosted regression trees is another ensemble method combining regression trees with weak individual predictive performances, into a single model with high performances [27,28].

First a regression tree model is fitted to a subset of data to minimize a loss function (in our case the deviance), which quantifies the loss in predictive performance due to a suboptimal model. The “boosting” algorithm is a numerical optimization technique for minimizing the loss function by iteratively fitting new trees to the prediction residuals of the preceding tree. For example, at the second step, a tree is fitted to the residuals of the first tree, and that second tree could contain different covariates and split nodes compared with the first. The two regression trees are combined and the residuals are calculated, a new tree is fitted and so on. To improve accuracy and reduce overfitting, each regression tree is grown from a bootstrap sample (without replacement) of the original dataset (usually 50% of the original sample) [29]. The final model is built by combining weighted individual tree contributions, weights being proportional to the trees performances.

To assess and rank potential associations with covariates, we used the Friedman “relative influence” [27], *i.e.*, the number of times a covariate is selected for splitting, weighted by the squared improvement of the loss function by splitting on that covariate. One thousand trees were used for each model. To allow complex interaction detection, the “interaction.depth” parameter of the BRT models was set to a value of 3; implying models with up to 3-way interactions.

#### Conventional logistic regression framework

Logistic regression (LR) is a well-known Generalized Linear Model adapted to test association between a binomial outcome and covariates [30]. In order to identify independent associations, we reproduced the usual epidemiologic analytic framework – that is, Univariate Followed by Multivariate Logistic Regression (UFMLR). We explored two thresholds for the selection of covariates in the univariate step, Pvalue <0.05 (UFMLR05) and Pvalue <0.20 (UFMLR20), with coefficient tested with the Wald test. We also distinguished whether or not a backward selection of covariates was further applied in multivariate regression model.

#### Least Absolute Shrinkage and Selection Operator logistic regression (LASSO)

We used LASSO to fit a multivariate logistic model with penalty on the magnitude of coefficients. The LASSO method maximizes the log-likelihood of the model, while applying constraints on the sum of the absolute values of the coefficients– shrinking the less important coefficients to zero [18]. The constraint was expressed in terms of a penalty parameter; the optimal value of this parameter was determined by minimizing the deviance of the multivariate logistic model (hereafter called LASSO-max) averaged over five-fold cross-validation subsamples [31]. In addition to this optimal model, we also considered a parsimonious LASSO model with a higher penalty parameter (hereafter called LASSO-se) so that the mean deviance of the model was within one standard error of the LASSO-max average deviance [28,32]. To rank potential associations with covariates, we used the proportion of estimated non-null parameters over one hundred LASSO models.

#### Permutation test for statistical significance of associations

No threshold exists to assess the statistical significance of measures of association, *i.e.* the Gini impurity criterion in RF models or the Friedman relative influence in BRT models or the proportion of estimated non-null parameters in LASSO models. We used permutation tests [33], which were applied in RF models to derive Pvalues for predictors and were suggested to be used with any method that provides a measure of covariate relevance [34]. The null hypothesis was the absence of association between a given covariate and the outcome. We computed the Pvalue by randomly permuting 999 times the values of the outcome and comparing the measures of association of the covariates in the permuted datasets with that from the original dataset [35]. Permutation of the outcome has the advantage of preserving the dependence structure between the covariates [34]. The one-sided Pvalue was computed as follows: $P_{x}=\frac{1+\mathrm{Number}\;\mathrm{of}\;\mathrm{permuted}\;\mathrm{datasets}\;\mathrm{where}\;S_{\mathrm{per}}>S_{\mathrm{obs}}}{1+\mathrm{Number}\;\mathrm{of}\;\mathrm{permutations}}$ with S_per_ and S_obs_ the measures of association for covariate *x* in the permuted and the real dataset, respectively. To increase consistency we also applied this method to UFMLR models, using the Wald test statistic as measure of association. We also checked whether permutation test Pvalues were similar to those obtained with the conventional Wald test (see Additional file 1).

### Simulated data

To assess the performances of the different methods in detection of associations in a similar-sized dataset, we simulated 500 datasets with 500 individuals and 300 covariates, sampled from a multivariate normal distribution (the simulation process is detailed in Additional file 1). The simulated logit model involved 150 continuous and 150 binary covariates. Four covariates of each type were directly associated with the outcome. Four covariates (2 binary and 2 continuous) were involved in pairwise multiplicative interactions. Two hundred and ninety-two covariates were not associated (neither directly nor through interactions) with the outcome. Twenty covariates (the 8 associated covariates and 12 non-associated covariates - 6 continuous and 6 binary) were correlated (ρ = 0.5). To explore the influence of correlation among a higher number of covariates, we increased the number of correlated covariates to 58 (the 8 associated covariates and 50 non-associated covariates – see Additional file 1). For both simulations, the number of permutations of the outcome was set to 99. Performances were assessed in terms of True Positive Rate (TPR), *i.e.* the proportion of associated covariates which were detected, and False Positive Rate (FPR), *i.e.* the proportion of non-associated covariates which were detected, at 5% nominal level (see Additional file 1 for TPR and FPR at 1% nominal level). We also distinguished between covariates with and without pairwise interactions for the calculation of TPR, and between covariates with and without correlations with associated covariates for the calculation of FPR.

### Fitting procedures and software

In all analyses, the covariates were centered and scaled prior to model fitting.

Statistical analyses were performed with R version 2.15.3. The following packages were used: “randomForest” [36] for RF models, “gbm” [37] for BRT models, “glmnet” [31] for LASSO-LR models.

## Results

### Detection of associations in the CoPanFlu dataset

With a nominal type I error of 5%, 11 independent associations were identified with RF, 12 with BRT, 24 with the LASSO-max, 8 with the LASSO-se, 3 to 5 with UFMLR05, and 9 to 10 with UFMLR20 (Table 1). Association between influenza infection and the pre-seasonal HAI titer was detected with all methods. The covariates “History of asthma”, “Professional activity involves contact with ill people”, “Daily frequency of hand washing (with soap or hand sanitizer) ≥ 5” and “Always or often covers mouth while coughing or sneezing”, were also identified by RF, BRT, and LASSO-max. This last covariate, as well as 23 additional covariates, were not detected by any of the UFMLR methods. Three associations identified by UFMLR20, with or without backward selection, were not retrieved by any of the data mining or LASSO methods. Of note, backward selection applied in UFMLR was associated with more and sometimes different associations than UFLMR without selection. Finally, 266 (87%) covariates were not selected by any method.

**Table 1:** Significant associations with RF, BRT, LASSO and UFMLR

	**RF**	**BRT**	**LASSO-max**	**LASSO-se**	**UFMLR05**	**UFMLR05 backward**	**UFMLR20**	**UFMLR20 backward**
**Covariates**								
Pre-seasonal HAI titer (log)	0.004	0.003	0.001	0.001	0.001	0.002	0.002	0.001
History of asthma^b^	0.009	0.012	0.001	0.001	0.009	0.019		0.004
Professional activity involves contact with ill people^b^	0.009	0.023	0.008				0.001	0.001
Age (years)		0.004	0.001	0.001		0.001		
Daily frequency of hand washing (with soap or hand sanitizer) ≥ 5^b^	0.014	0.023	0.019					0.008
Always or often covers mouth while coughing or sneezing^b^	0.033	0.023	0.003	0.001				
“Craftsman, shopkeeper, chief executive officer” (socio-professional group)^b^		0.036	0.006				0.047	
History of chemotherapy^b^	0.048		0.021				0.027	
Average living room temperature (°C)			0.001	0.001		0.031	0.047	0.002
Presence of a dishwasher in the kitchen^b^			0.002	0.001	0.048	0.007		0.007
Sex = male^b^	0.048							
Professional activity is primarily outdoors^b^	0.045							
Age < 15 years^b^	0.016							
Any respiratory disease^b^	0.030							
Number of children (<15 years) in the household (n)	0.004							
Body mass index (kg/m2)		0.032						
Proportion of inhabitants > 15 years without a diploma (in IRIS zone^i^)		0.024						
Proportion of habitations rented by inhabitants (in IRIS zone^i^)		0.023						
Proportion of habitations owned by inhabitants (in IRIS zone^i^)		0.049						
Habitation = house^b^		0.016						
Duration of contacts with subjects aged between 60 and 99 years (log (min))			0.006				0.023	0.019
Number of subjects in the household (n)			0.002	0.002				
Kitchen surface area per subject (m2)			0.005	0.002				
Cardiac arrhythmia^b^			0.021					
History of radiotherapy^b^			0.016					
Daily consumption of green tea (n)			0.040					
Number of birds inside habitation (n)			0.030					
Number of rooms per subject in habitation (n)			0.009					
Number of children in the bedroom (n)			0.010					
Bedroom windows face: garden^b^			0.010					
Duration of contacts at home (log (min))			0.029					
Longitude of the habitation (degrees)			0.027					
Latitude of the habitation (degrees)			0.028					
Proportion of “farmer, primary sector” (socio-professional group) (in IRIS zone^i^)			0.004					0.005
Kitchen filtration of area^b^							0.021	0.034
Tiles flooring in the kitchen^b^							0.015	0.029
Agricultural land near habitation^b^							0.048	

^*b*^binary covariate. ^i^IRIS zones are statistical block groups of about 2000 inhabitants defined by the French Institut National de la Statistique et des Etudes Economiques (INSEE).

### Simulated data

**Figure 1:**
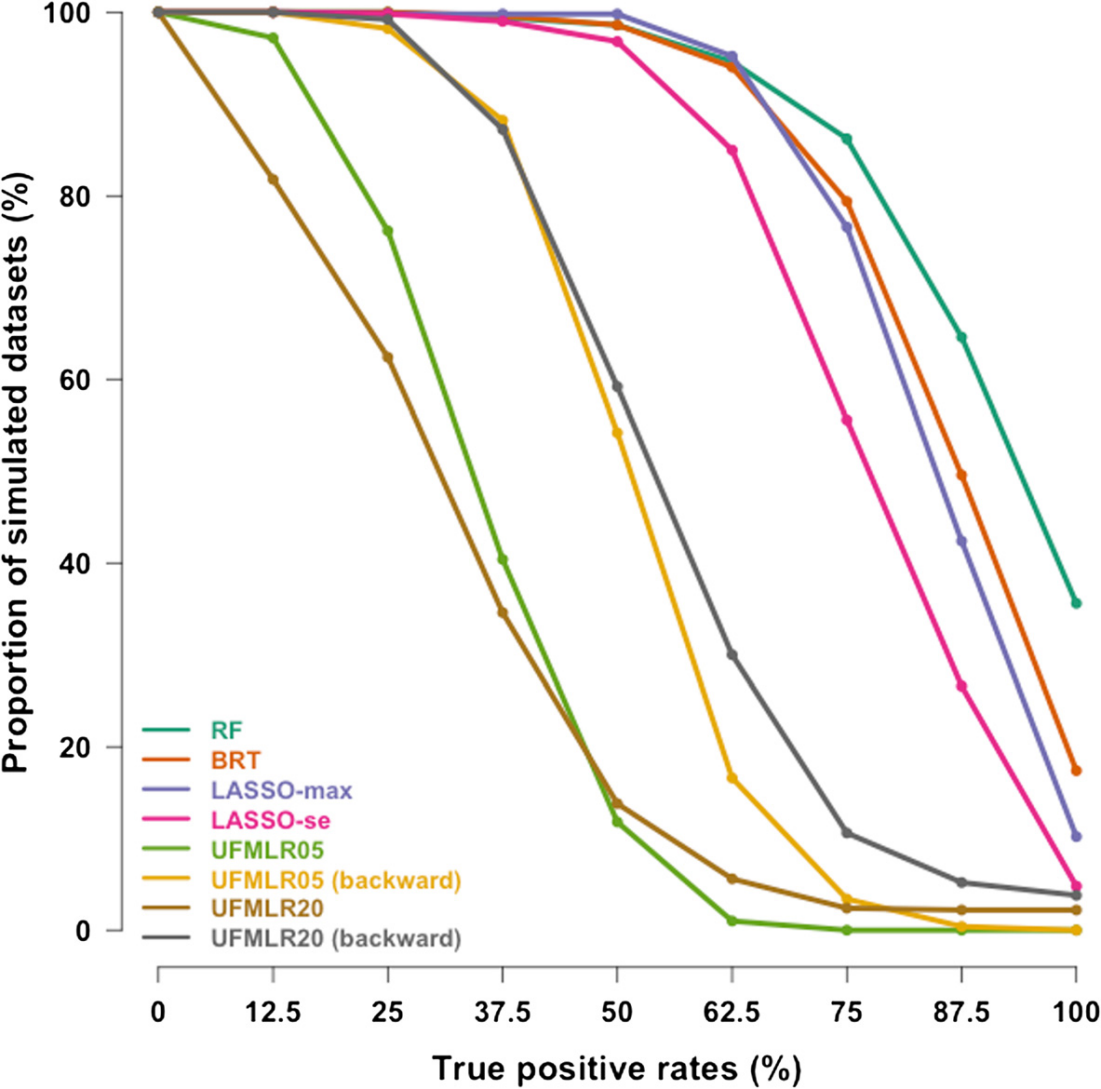
**Cumulative distribution curves of the True Positive Rates in the 500 simulated datasets.** y-axis shows the proportion of simulated datasets with True Positive Rates above or equal to the True Positive Rates on the x-axis.

**Figure 2:**
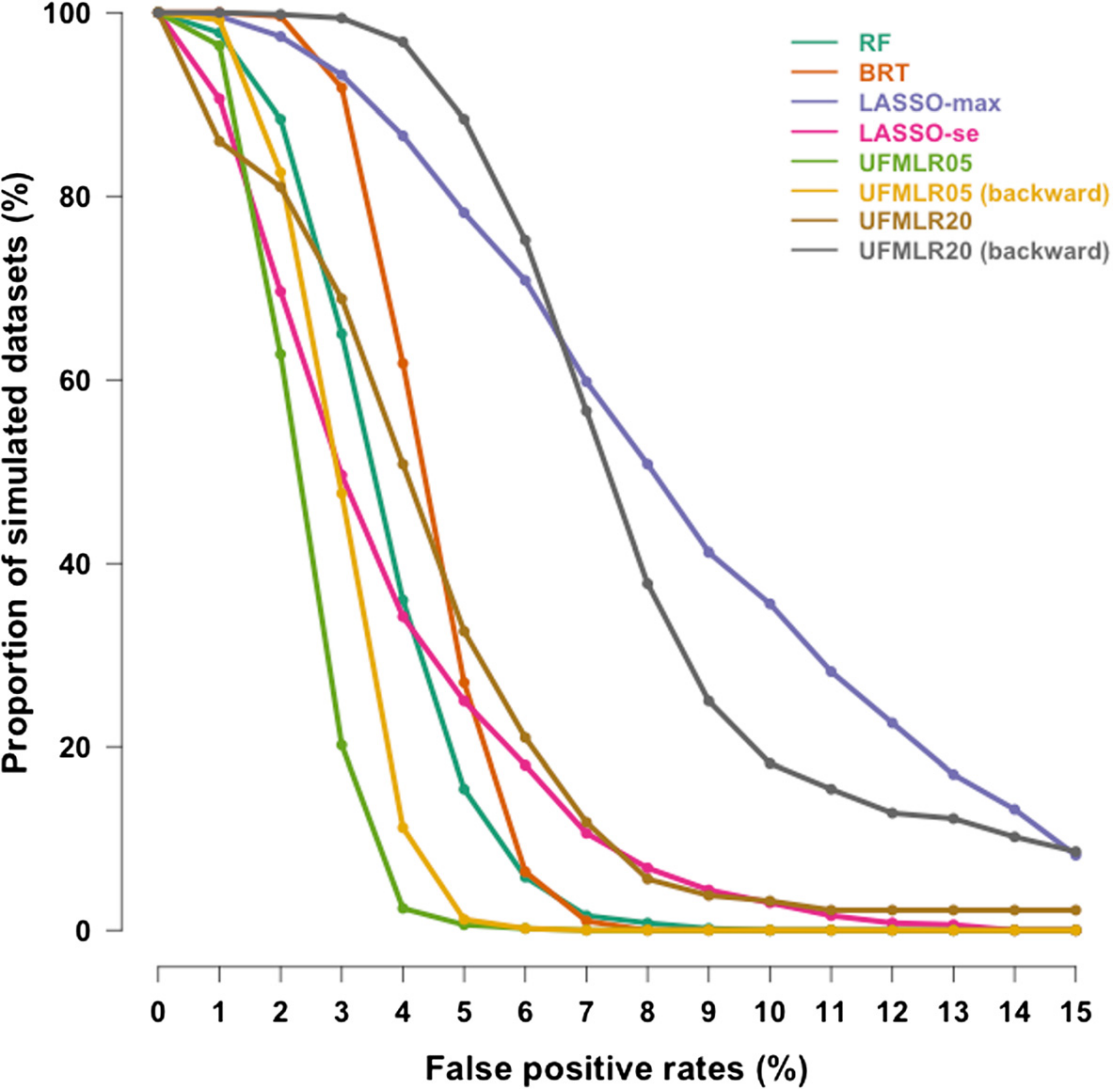
**Cumulative distribution curves of the False Positive Rates in the 500 simulated datasets.** y-axis shows the proportion of simulated datasets with False Positive Rates above or equal to the False Positive Rates on the x-axis.

At 5% nominal level, the True Positive Rate was 85% with RF, 80% with BRT and 71% to 78% with LASSO (Table 2). The UFMLR, with or without backward selection, was the least efficient method at detecting true associations, with a TPR ranging between 26% and 49%. All methods, except RF and BRT, exhibited higher TPR when associated covariates did not interact with other associated covariates. The TPR for continuous covariates was higher than the TPR for binary covariates with all methods. The proportion of simulated datasets in which all associated covariates were detected was 36% with RF, 17% for BRT, 10% for LASSO-max, 5% for LASSO-se, 0% for UFMLR05 with or without backward selection, 4% and 2% for UFMLR20 with and without backward selection, respectively (Figure 1). UFMLR20 without backward selection detected none of the associated covariates in 18% of the simulated datasets. Overall, the FPR was below or equal to the nominal type I error with all methods except for LASSO-max and UFMLR20 with backward selection. UFLMR05 without backward selection was the most conservative with a FPR of 2% (Table 2 and Figure 2). RF, BRT, LASSO methods and UFMLR20 with backward selection suffered from an increase of type I error when the non-associated covariates were correlated with associated covariates.

**Table 2:** Performances of RF, BRT, LASSO and UFMLR in the 500 simulated datasets

	**n**	**RF**	**BRT**	**LASSO-max**	**LASSO-se**	**UFMLR05**	**UFMLR05 backward**	**UFMLR20**	**UFMLR20 backward**
**Type I error 5%**									
True Positive Rates (TPR)	8	85% (55% - 100%)	80% (51% - 100%)	78% (52% - 100%)	71% (41% - 100%)	28% (3% - 54%)	45% (20% - 70%)	26% (0% - 65%)	49% (15% - 84%)
*Covariates with pairwise interaction*	4	86% (49% - 100%)	80% (41% - 100%)	77% (40% - 100%)	69% (26% - 100%)	24% (0% - 63%)	41% (0% - 83%)	24% (0% - 72%)	46% (0% - 96%)
*Covariates without pairwise interaction*	4	84% (46% - 100%)	79% (41% - 100%)	79% (41% - 100%)	73% (32% - 100%)	32% (0% - 74%)	50% (6% - 93%)	28% (0% - 76%)	53% (5% - 100%)
*Continuous covariates*	4	90% (55% - 100%)	82% (47% - 100%)	82% (49% - 100%)	77% (41% - 100%)	35% (0% - 74%)	49% (15% - 84%)	29% (0% - 76%)	55% (14% - 95%)
*Binary covariates*	4	80% (34% - 100%)	78% (35% - 100%)	74% (34% - 100%)	64% (20% - 100%)	22% (0% - 57%)	41% (8% - 74%)	23% (0% - 69%)	44% (0% - 90%)
False Positive Rates (FPR)	292	4% (1% - 6%)	4% (2% - 6%)	9% (0% - 17%)	4% (0% - 9%)	2% (1% - 4%)	3% (1% - 5%)	4% (0% - 12%)	9% (0% - 18%)
*Covariates correlated with associated covariates*	12	46% (7% - 85%)	33% (3% - 63%)	23% (0% - 48%)	18% (0% - 41%)	4% (0% - 15%)	7% (0% - 22%)	9% (0% - 39%)	19% (0% - 60%)
*Covariates uncorrelated with associated covariates*	280	2% (0% - 4%)	3% (1% - 5%)	8% (0% - 17%)	3% (0% - 8%)	2% (1% - 4%)	3% (1% - 4%)	4% (0% - 11%)	8% (0% - 16%)
*Continuous covariates*	146	3% (0% - 6%)	3% (1% - 6%)	9% (0% - 18%)	4% (0% - 9%)	2% (0% - 5%)	3% (0% - 6%)	5% (0% - 13%)	9% (0% - 18%)
*Binary covariates*	146	4% (0% - 8%)	5% (2% - 9%)	9% (0% - 18%)	3% (0% - 9%)	2% (0% - 5%)	3% (0% - 5%)	4% (0% - 12%)	9% (0% - 18%)

Performances are shown as: Mean (95% confidence interval).

When increasing the number of non-associated covariates correlated with associated covariates, the TPR decreased with all methods, except for UFMLR20 with backward selection. The FPR were close to the FPR observed with a lower number of non-associated correlated covariates, with the exception of UFMLR20 with backward selection (14%, see Additional file 1).

## Discussion

Without any pre-specified hypotheses, Random Forests, Boosted regression trees and LASSO models identified 8 to 24 covariates independently associated with influenza infection, among which 23 were not detected by the “univariate followed by multivariate logistic regression” framework. On the other hand, when a Pvalue threshold of 0.20 was applied to select covariates for multivariate logistic regression during univariate logistic models, a substantial number of spurious independent associations were detected which were not retrieved by any other methods. Simulations showed that RF, BRT and LASSO outperformed the conventional logistic framework to detect independent associations, while the false positive detection rates remained at the nominal significance level (RF, BRT and LASSO-se) or moderately increased above it (LASSO-max).

When covariates not associated with the outcome were correlated with covariates associated with the outcome, the false positive rate was high, particularly with RF. For this method, this finding was explained by the sensitivity of the Gini impurity criterion to between-covariates correlation [38]. More strikingly, increasing the number of correlated covariates also affected the true positive rate, which decreased with almost all methods (see Additional file 1). This finding may be attributed to a decrease of covariate strength of association due to a large number of correlated covariates and consequently, a decrease to be detected by any of the methods, as was shown in RF and LASSO [39].

In this work, we used an exploratory approach to analyze a large epidemiologic dataset, *i.e.* we aimed to detect associations between numerous covariates and an outcome, without pre-specified hypothesis. Despite the high number of covariates under study, the multiple testing issue was not considered. It is common to distinguish between two type of error rates: the comparisonwise error rate (CER) which corresponds to the probability, for an individual test, to reject the null hypothesis when it is actually true; and the experimentwise error rate (EER - also known as the familywise error rate), which corresponds to the probability of rejecting at least one true null hypothesis among the multiple tests performed [40]. According to the simulations performed, we observed that the false positive rates associated to the permutation test was close to the expected CER level (5%) with almost all methods. Working at the individual covariate level, no adjustment was necessary. Adjusting Pvalues would have been required if the EER had to be controlled, *e.g.* in order to build a predictive model or to confirm the detected associations [41]. It is nevertheless essential to keep in mind that the significant results correspond to exploratory results, which require further confirmation.

To our knowledge, our study is the first to compare the performance in terms of associations detection of the random forest and boosted regression trees importance measures to the LASSO and the widely used analytic framework in the simultaneous analysis of hundreds to thousands of covariates to detect independent associations, a growing issue in epidemiology. Although such datasets offer analytic challenges, they are hardly comparable to datasets explored in omic-based approaches, in which the number of covariates (up to millions) is far higher to the number of samples, and for which the use of dedicated approaches, *e.g.* the elastic net penalty [42], would have been unavoidable.

Some associations with influenza infection detected with RF, BRT or LASSO-se methods were expected: HAI titers are well-known correlates of protection against influenza infection [43], young age is a known risk factor for H1N1pdm influenza infection [44], non-pharmaceutical preventive measures such as handwashing have been found to be determinants of H1N1pdm infection [45], and asthma was also reported as a specific risk factor [46]. Having a professional activity involving contact with ill people sounds logical as a potential risk factor, and several reports have shown that hospital staff were at increased risk of infection [47]*.* For other associations, *e.g.* “Always or often covers mouth while coughing or sneezing”, we did not find consistent findings in the literature and it could be hard to hypothesize how the detected covariates could be linked with the risk of H1N1pdm influenza infection. However, “Professional activity involves contact with ill people” and age were correlated with this covariate (ρ = 0.10, Pvalue = 0.020 and ρ = 0.29, Pvalue < 0.001, respectively); based on our simulation findings we suspect that this association, as many others (*e.g.* “Presence of a dishwasher in the kitchen”), are likely to be false positives.

Having no prior knowledge about the covariates truly associated with influenza infections we performed a simulation study to assess the performances of the different methods at detecting true and false associations in similar sized data, with a similar number of positive outcomes and covariates. Although we did not perform an extensive analysis exploring varying proportions of associated covariates or interactions between covariates, our simulations clearly demonstrated that UFMLR, with or without backward selection, were inefficient. We developed permutation tests to assess the significance of the covariates association with the outcome in RF, BRT and LASSO; their results with UFMLR were comparable to that of the Wald test in terms of nominal coverage (see Additional file 1). Although permutation tests exhibited slightly less power than the Wald test, this did not modify our general findings.

## Conclusions

The conventional multivariate logistic regression framework is obviously not adapted for exploratory analysis of large epidemiologic datasets in view of detecting independent associations without any pre-specified hypothesis. In this respect, data mining methods and LASSO should be considered as credible alternatives to multivariate logistic regression.

## Abbreviations

LASSO: Least absolute shrinkage and selection operator; H1N1pdm influenza: H1N1 pandemic influenza; HAI: Haemagglutination inhibition; RF: Random forests; BRT: Boosted regression trees; LR: Logistic regression; UFMLR: Univariate followed by multivariate logistic regression; TPR: True positive rate; FPR: False positive rate.

## Competing interests

The authors declare that they have no competing interests.

## Authors’ contributions

YM and FC conceived and designed the experiments. YM performed the experiments and analyzed the data. YM and FC wrote the paper. All authors read and approved the final manuscript.

## Pre-publication history

The pre-publication history for this paper can be accessed here:

http://www.biomedcentral.com/1471-2288/14/99/prepub

## Supplementary Material

Additional file 1 **Simulation process description and additional simulation results.** Description of the simulation process; TPR and FPR at 1% nominal significance level; TPR and FPR with an increased number of correlated covariates; and comparisons of UFMLR TPR and FPR with Wald test and permutation test. Click here for file
