# Supplementary material for: Detection of independent associations in a large epidemiologic dataset: a comparison of random forests, boosted regression trees, conventional and penalized logistic regression for identifying independent factors associated with H1N1pdm influenza infections
Source: BMC Med Res Methodol. 2014 Aug 26;14:99. doi: 10.1186/1471-2288-14-99 (PMC4146451; doi:10.1186/1471-2288-14-99)
Supplement: Additional file 1 — Simulation process description and additional simulation results. Description of the simulation process; TPR and FPR at 1% nominal significance level; TPR and FPR with an increased number of correlated covariates; and comparisons of UFMLR TPR and FPR with Wald test and permutation test. [file 1471-2288-14-99-S1.pdf]

Additional file 1

Simulated data

Five hundred datasets were sampled from a multivariate normal distribution with standard normal marginal (the simulation process is summarized in Table 1). We simulated a binary outcome using the following logit model:

$$\log\left(\frac{p_i}{1-p_i}\right) =$$

$$\mu + \sum_{j=1}^{C} \beta cont_j c_{i,j} + \sum_{k=1}^{B} \beta bin_k b_{i,k} + \sum_{l=1}^{C} \sum_{m=1}^{C} \eta cont_{lm} c_{i,l} c_{i,m} + \sum_{n=1}^{B} \sum_{o=1}^{B} \eta bin_{no} b_{i,n} b_{i,o} + \varepsilon$$

with C (C=150) the number of continuous covariates *c* and B (B=150) the number of binary covariates *b*. To mimic binary covariates, sampled continuous covariates were set equal to 0 or 1 depending on whether the sampled values were lower or higher than the median of the standard normal distribution. The interaction parameters $\eta cont_{kl}$ and ηbin_{kl}were set equal to 0, except for the special cases $\eta cont_{12}$= ηbin_{12}= 0.5. The sample size was 500 and the mean proportion of outcome in the 500 simulated datasets was 14% (95% CI = [11%;18%]).

Two scenarios were simulated, in scenario 1 (whose results are detailed in the manuscript) twenty covariates (the 8 associated covariates and 12 non-associated covariates - 6 continuous and 6 binary) were correlated (ρ=0.5). In scenario 2, we increased the number of correlated covariates to 58 (the 8 associated covariates and 50 non-associated covariates).

Tables

Table 1 Summary of the simulation process

**Scenario**	Scenario 1	Scenario 2
**Number of continuous covariates (C)**	150	150
**Number of binary covariates (B)**	150	150
**Number of covariates with direct effects**	8	8
**Number of covariates with interaction effects**	4	4
**Number of non-associated covariates**	292	292
**Intercept**	$\mu = 1.5$	$\mu = 1.5$
**Direct effects**	$\beta cont_i = \beta bin_i$ $= \begin{cases} 0.5\ if\ i\ \in \{1:4\} \\ 0\ otherwise \end{cases}$	$\beta cont_i = \beta bin_i$ $= \begin{cases} 0.5\ if\ i\ \in \{1:4\} \\ 0\ otherwise \end{cases}$
**Gaussian noise**	$\varepsilon \sim \mathcal{N}(0,1)$	$\varepsilon \sim \mathcal{N}(0,1)$
**Covariances**	$Cov(x_{i,j}, x_{i,k}) =$ $\begin{cases} 1\ if\ i = j \\ 0.5\ if\ i,j\ \in \{1:10\} \\ 0\ otherwise \end{cases}$ with x either a binary or a continuous covariate	$Cov(x_{i,j}, x_{i,k}) =$ $\begin{cases} 1\ if\ i = j \\ 0.5\ if\ i,j\ \in \{1:29\} \\ 0\ otherwise \end{cases}$ with x either a binary or a continuous covariate

Table 2. Performances of RF, BRT, LASSO and UFMLR in the 500 simulated datasets (Type I error 1%) – Scenario 1. Performances are shown as: Mean (95% confidence interval)

	**n**	**RF**	**BRT**	**LASSO-max**	**LASSO-se**	**UFMLR05**	**UFMLR05 backward**	**UFMLR20**	**UFMLR20 backward**
**Type I error 1%**									
True Positive Rates (TPR)	8	58% (17% - 99%)	53% (20% - 86%)	72% (44% - 100%)	71% (41% - 100%)	11% (0% - 31%)	29% (6% - 53%)	11% (0% - 44%)	38% (1% - 75%)
Covariates with pairwise interaction	4	60% (9% - 100%)	54% (7% - 100%)	71% (30% - 100%)	69% (25% - 100%)	9% (0% - 36%)	25% (0% - 61%)	10% (0% - 46%)	35% (0% - 84%)
Covariates without pairwise interaction	4	56% (8% - 100%)	53% (9% - 96%)	74% (34% - 100%)	73% (32% - 100%)	13% (0% - 44%)	34% (0% - 75%)	12% (0% - 52%)	42% (0% - 90%)
Continuous covariates	4	71% (14% - 100%)	66% (24% - 100%)	79% (44% - 100%)	77% (41% - 100%)	15% (0% - 46%)	34% (0% - 69%)	12% (0% - 51%)	43% (0% - 86%)
Binary covariates	4	45% (0% - 96%)	40% (0% - 89%)	66% (23% - 100%)	64% (20% - 100%)	7% (0% - 31%)	25% (0% - 59%)	9% (0% - 46%)	33% (0% - 80%)
False Positive Rates (FPR)	292	1% (0% - 2%)	1% (0% - 2%)	4% (0% - 9%)	3% (0% - 8%)	1% (0% - 2%)	1% (0% - 3%)	2% (0% - 9%)	5% (0% - 15%)
Covariates correlated with associated covariates	12	18% (0% - 45%)	12% (0% - 31%)	20% (0% - 43%)	18% (0% - 41%)	1% (0% - 5%)	3% (0% - 13%)	3% (0% - 31%)	13% (0% - 52%)
Covariates uncorrelated with associated covariates	280	0% (0% - 1%)	1% (0% - 2%)	3% (0% - 8%)	3% (0% - 8%)	1% (0% - 2%)	1% (0% - 2%)	1% (0% - 8%)	5% (0% - 14%)
Continuous covariates	146	1% (0% - 3%)	1% (0% - 2%)	4% (0% - 10%)	4% (0% - 9%)	1% (0% - 2%)	1% (0% - 3%)	2% (0% - 9%)	5% (0% - 16%)
Binary covariates	146	1% (0% - 3%)	1% (0% - 4%)	4% (0% - 9%)	3% (0% - 8%)	1% (0% - 2%)	1% (0% - 3%)	1% (0% - 9%)	5% (0% - 16%)

Table 3 Performances of RF, BRT, LASSO and UFMLR in the 500 simulated datasets (Type I error 5%) – Scenario 2. Performances are shown as: Mean (95% confidence interval)

	n	RF	BRT	LASSO-max	LASSO-se	UFMLR05	UFMLR05 backward	UFMLR20	UFMLR20 backward
**Type I error 5%**									
True Positive Rates (TPR)	8	68% (30% - 100%)	72% (40% - 100%)	70% (41% - 98%)	63% (33% - 93%)	27% (0% - 56%)	40% (12% - 67%)	16% (0% - 70%)	56% (10% - 100%)
Covariates with pairwise interaction	4	60% (12% - 100%)	67% (22% - 100%)	67% (25% - 100%)	60% (13% - 100%)	23% (0% - 64%)	35% (0% - 77%)	15% (0% - 71%)	54% (0% - 100%)
Covariates without pairwise interaction	4	77% (31% - 100%)	77% (35% - 100%)	72% (32% - 100%)	66% (26% - 100%)	31% (0% - 74%)	44% (1% - 87%)	18% (0% - 75%)	58% (4% - 100%)
Continuous covariates	4	63% (13% - 100%)	70% (29% - 100%)	73% (36% - 100%)	68% (29% - 100%)	33% (0% - 75%)	45% (6% - 83%)	18% (0% - 76%)	60% (8% - 100%)
Binary covariates	4	74% (23% - 100%)	74% (27% - 100%)	66% (24% - 100%)	58% (15% - 100%)	20% (0% - 58%)	34% (0% - 72%)	14% (0% - 68%)	52% (0% - 100%)
False Positive Rates (FPR)	292	5% (1% - 10%)	6% (4% - 9%)	9% (1% - 17%)	4% (0% - 9%)	2% (0% - 5%)	3% (1% - 5%)	4% (0% - 20%)	14% (0% - 31%)
Covariates correlated with associated covariates	50	26% (1% - 50%)	25% (10% - 41%)	16% (5% - 26%)	12% (3% - 21%)	4% (0% - 11%)	6% (0% - 13%)	9% (0% - 56%)	33% (0% - 84%)
Covariates uncorrelated with associated covariates	242	1% (0% - 2%)	3% (1% - 5%)	8% (0% - 16%)	3% (0% - 8%)	2% (0% - 4%)	3% (1% - 5%)	3% (0% - 13%)	11% (1% - 20%)
Continuous covariates	146	4% (0% - 9%)	5% (2% - 8%)	9% (1% - 18%)	5% (0% - 10%)	3% (0% - 6%)	4% (1% - 7%)	4% (0% - 21%)	15% (0% - 32%)
Binary covariates	146	6% (0% - 12%)	8% (3% - 13%)	9% (1% - 17%)	4% (0% - 9%)	2% (0% - 5%)	3% (0% - 6%)	4% (0% - 19%)	14% (0% - 30%)

Table 4. Performances of RF, BRT, LASSO and UFMLR in the 500 simulated datasets (Type I error 1%) – Scenario 2. Performances are shown as: Mean (95% confidence interval)

	n	RF	BRT	LASSO-max	LASSO-se	UFMLR05	UFMLR05 backward	UFMLR20	UFMLR20 backward
**Type I error 1%**									
True Positive Rates (TPR)	8	40% (2% - 78%)	53% (20% - 87%)	65% (35% - 94%)	62% (32% - 92%)	10% (0% - 32%)	27% (2% - 51%)	10% (0% - 60%)	44% (0% - 99%)
Covariates with pairwise interaction	4	28% (0% - 73%)	44% (0% - 93%)	60% (15% - 100%)	59% (12% - 100%)	7% (0% - 32%)	22% (0% - 59%)	9% (0% - 60%)	42% (0% - 100%)
Covariates without pairwise interaction	4	52% (0% - 100%)	63% (17% - 100%)	69% (29% - 100%)	66% (25% - 100%)	14% (0% - 47%)	31% (0% - 70%)	11% (0% - 62%)	47% (0% - 100%)
Continuous covariates	4	36% (0% - 81%)	55% (13% - 97%)	71% (33% - 100%)	68% (29% - 100%)	14% (0% - 48%)	30% (0% - 65%)	11% (0% - 63%)	48% (0% - 100%)
Binary covariates	4	44% (0% - 100%)	51% (1% - 100%)	58% (14% - 100%)	56% (13% - 100%)	6% (0% - 30%)	23% (0% - 56%)	9% (0% - 59%)	41% (0% - 100%)
False Positive Rates (FPR)	292	2% (0% - 4%)	2% (0% - 4%)	5% (0% - 10%)	4% (0% - 9%)	1% (0% - 2%)	1% (0% - 3%)	3% (0% - 19%)	11% (0% - 30%)
Covariates correlated with associated covariates	50	11% (0% - 25%)	10% (0% - 20%)	13% (4% - 23%)	12% (3% - 21%)	1% (0% - 4%)	2% (0% - 7%)	7% (0% - 54%)	25% (0% - 80%)
Covariates uncorrelated with associated covariates	242	0% (0% - 1%)	0% (0% - 1%)	4% (0% - 9%)	3% (0% - 7%)	1% (0% - 2%)	1% (0% - 3%)	2% (0% - 12%)	8% (0% - 19%)
Continuous covariates	146	2% (0% - 5%)	2% (0% - 4%)	6% (0% - 11%)	4% (0% - 10%)	1% (0% - 2%)	2% (0% - 4%)	3% (0% - 20%)	11% (0% - 31%)
Binary covariates	146	2% (0% - 6%)	2% (0% - 5%)	5% (0% - 10%)	4% (0% - 9%)	1% (0% - 2%)	1% (0% - 3%)	3% (0% - 18%)	10% (0% - 29%)

Table 5 Performances of UFMLR in the 500 simulated datasets (Type I error 5%) – Scenario 1. [w] Wald test Pvalue. [p] permutation test Pvalue. Performances are shown as: Mean (95% confidence interval)

	n	UFMLR05 [w]	UFMLR05 [p]	UFMLR05 backward [w]	UFMLR05 backward [p]	UFMLR20 [w]	UFMLR20 [p]	UFMLR20 backward [w]	UFMLR20 backward [p]
**Type I error 5%**									
True Positive Rates (TPR)	8	31% (5% - 58%)	28% (3% - 54%)	47% (22% - 72%)	45% (20% - 70%)	32% (0% - 74%)	26% (0% - 65%)	51% (16% - 86%)	49% (15% - 84%)
Covariates with pairwise interaction	4	27% (0% - 66%)	24% (0% - 63%)	43% (0% - 86%)	41% (0% - 83%)	30% (0% - 81%)	24% (0% - 72%)	47% (0% - 97%)	46% (0% - 96%)
Covariates without pairwise interaction	4	36% (0% - 79%)	32% (0% - 74%)	51% (8% - 94%)	50% (6% - 93%)	34% (0% - 86%)	28% (0% - 76%)	55% (7% - 100%)	53% (5% - 100%)
Continuous covariates	4	38% (0% - 80%)	35% (0% - 74%)	51% (17% - 86%)	49% (15% - 84%)	35% (0% - 87%)	29% (0% - 76%)	56% (16% - 97%)	55% (14% - 95%)
Binary covariates	4	24% (0% - 61%)	22% (0% - 57%)	43% (9% - 76%)	41% (8% - 74%)	29% (0% - 78%)	23% (0% - 69%)	46% (0% - 92%)	44% (0% - 90%)
False Positive Rates (FPR)	292	3% (1% - 4%)	2% (1% - 4%)	3% (1% - 5%)	3% (1% - 5%)	6% (0% - 14%)	4% (0% - 12%)	9% (0% - 18%)	9% (0% - 18%)
Covariates correlated with associated covariates	12	4% (0% - 16%)	4% (0% - 15%)	8% (0% - 23%)	7% (0% - 22%)	12% (0% - 44%)	9% (0% - 39%)	20% (0% - 61%)	19% (0% - 60%)
Covariates uncorrelated with associated covariates	280	2% (1% - 4%)	2% (1% - 4%)	3% (1% - 5%)	3% (1% - 4%)	6% (0% - 13%)	4% (0% - 11%)	9% (1% - 16%)	8% (0% - 16%)
Continuous covariates	146	3% (0% - 5%)	2% (0% - 5%)	3% (0% - 6%)	3% (0% - 6%)	6% (0% - 15%)	5% (0% - 13%)	9% (0% - 19%)	9% (0% - 18%)
Binary covariates	146	2% (0% - 5%)	2% (0% - 5%)	3% (0% - 6%)	3% (0% - 5%)	6% (0% - 15%)	4% (0% - 12%)	9% (0% - 19%)	9% (0% - 18%)

Table 6 Performances of UFMLR in the 500 simulated datasets (Type I error 1%) – Scenario 1. [w] Wald test Pvalue. [p] permutation test Pvalue. Performances are shown as: Mean (95% confidence interval)

	n	**UFMLR05 [w]**	**UFMLR05 [p]**	**UFMLR05 backward [w]**	**UFMLR05 backward [p]**	**UFMLR20 [w]**	**UFMLR20 [p]**	**UFMLR20 backward [w]**	**UFMLR20 backward [p]**
**Type I error 1%**									
True Positive Rates (TPR)	8	14% (0% - 36%)	11% (0% - 31%)	34% (10% - 58%)	29% (6% - 53%)	17% (0% - 54%)	11% (0% - 44%)	43% (7% - 78%)	38% (1% - 75%)
Covariates with pairwise interaction	4	11% (0% - 40%)	9% (0% - 36%)	29% (0% - 66%)	25% (0% - 61%)	16% (0% - 59%)	10% (0% - 46%)	39% (0% - 89%)	35% (0% - 84%)
Covariates without pairwise interaction	4	17% (0% - 50%)	13% (0% - 44%)	39% (0% - 81%)	34% (0% - 75%)	19% (0% - 63%)	12% (0% - 52%)	46% (0% - 95%)	42% (0% - 90%)
Continuous covariates	4	18% (0% - 53%)	15% (0% - 46%)	39% (5% - 73%)	34% (0% - 69%)	19% (0% - 63%)	12% (0% - 51%)	48% (8% - 88%)	43% (0% - 86%)
Binary covariates	4	9% (0% - 37%)	7% (0% - 31%)	29% (0% - 61%)	25% (0% - 59%)	15% (0% - 57%)	9% (0% - 46%)	37% (0% - 84%)	33% (0% - 80%)
False Positive Rates (FPR)	292	1% (0% - 2%)	1% (0% - 2%)	1% (0% - 3%)	1% (0% - 3%)	3% (0% - 10%)	2% (0% - 9%)	6% (0% - 16%)	5% (0% - 15%)
Covariates correlated with associated covariates	12	1% (0% - 6%)	1% (0% - 5%)	4% (0% - 14%)	3% (0% - 13%)	5% (0% - 33%)	3% (0% - 31%)	15% (0% - 55%)	13% (0% - 52%)
Covariates uncorrelated with associated covariates	280	1% (0% - 2%)	1% (0% - 2%)	1% (0% - 3%)	1% (0% - 2%)	3% (0% - 9%)	1% (0% - 8%)	6% (0% - 15%)	5% (0% - 14%)
Continuous covariates	146	1% (0% - 3%)	1% (0% - 2%)	2% (0% - 4%)	1% (0% - 3%)	3% (0% - 11%)	2% (0% - 9%)	6% (0% - 17%)	5% (0% - 16%)
Binary covariates	146	1% (0% - 2%)	1% (0% - 2%)	1% (0% - 3%)	1% (0% - 3%)	3% (0% - 10%)	1% (0% - 9%)	6% (0% - 17%)	5% (0% - 16%)

Table 7 Performances of UFMLR in the 500 simulated datasets (Type I error 5%) – Scenario 2. [w] Wald test Pvalue. [p] permutation test Pvalue. Performances are shown as: Mean (95% confidence interval)

	n	UFMLR05 [w]	UFMLR05 [p]	UFMLR05 backward [w]	UFMLR05 backward [p]	UFMLR20 [w]	UFMLR20 [p]	UFMLR20 backward [w]	UFMLR20 backward [p]
**Type I error 5%**									
True Positive Rates (TPR)	8	33% (3% - 63%)	27% (0% - 56%)	41% (14% - 69%)	40% (12% - 67%)	20% (0% - 79%)	16% (0% - 70%)	57% (12% - 100%)	56% (10% - 100%)
Covariates with pairwise interaction	4	30% (0% - 73%)	23% (0% - 64%)	38% (0% - 81%)	35% (0% - 77%)	19% (0% - 80%)	15% (0% - 71%)	55% (0% - 100%)	54% (0% - 100%)
Covariates without pairwise interaction	4	36% (0% - 80%)	31% (0% - 74%)	45% (2% - 89%)	44% (1% - 87%)	22% (0% - 85%)	18% (0% - 75%)	59% (6% - 100%)	58% (4% - 100%)
Continuous covariates	4	41% (0% - 85%)	33% (0% - 75%)	47% (8% - 86%)	45% (6% - 83%)	23% (0% - 87%)	18% (0% - 76%)	61% (9% - 100%)	60% (8% - 100%)
Binary covariates	4	25% (0% - 66%)	20% (0% - 58%)	36% (0% - 74%)	34% (0% - 72%)	18% (0% - 76%)	14% (0% - 68%)	53% (0% - 100%)	52% (0% - 100%)
False Positive Rates (FPR)	292	3% (1% - 6%)	2% (0% - 5%)	4% (1% - 6%)	3% (1% - 5%)	5% (0% - 22%)	4% (0% - 20%)	15% (0% - 31%)	14% (0% - 31%)
Covariates correlated with associated covariates	50	6% (0% - 15%)	4% (0% - 11%)	7% (0% - 14%)	6% (0% - 13%)	12% (0% - 59%)	9% (0% - 56%)	34% (0% - 85%)	33% (0% - 84%)
Covariates uncorrelated with associated covariates	242	3% (1% - 5%)	2% (0% - 4%)	3% (1% - 5%)	3% (1% - 5%)	4% (0% - 15%)	3% (0% - 13%)	11% (2% - 20%)	11% (1% - 20%)
Continuous covariates	146	4% (0% - 7%)	3% (0% - 6%)	4% (1% - 7%)	4% (1% - 7%)	6% (0% - 23%)	4% (0% - 21%)	16% (0% - 32%)	15% (0% - 32%)
Binary covariates	146	3% (0% - 6%)	2% (0% - 5%)	3% (0% - 6%)	3% (0% - 6%)	5% (0% - 21%)	4% (0% - 19%)	14% (0% - 30%)	14% (0% - 30%)

Table 8 Performances of UFMLR in the 500 simulated datasets (Type I error 1%) – Scenario 2. [w] Wald test Pvalue. [p] permutation test Pvalue. Performances are shown as: Mean (95% confidence interval)

	n	UFMLR05 [w]	UFMLR05 [p]	UFMLR05 backward [w]	UFMLR05 backward [p]	UFMLR20 [w]	UFMLR20 [p]	UFMLR20 backward [w]	UFMLR20 backward [p]
**Type I error 1%**									
True Positive Rates (TPR)	8	16% (0% - 41%)	10% (0% - 32%)	30% (6% - 55%)	27% (2% - 51%)	14% (0% - 66%)	10% (0% - 60%)	51% (1% - 100%)	44% (0% - 99%)
Covariates with pairwise interaction	4	12% (0% - 44%)	7% (0% - 32%)	26% (0% - 64%)	22% (0% - 59%)	13% (0% - 67%)	9% (0% - 60%)	49% (0% - 100%)	42% (0% - 100%)
Covariates without pairwise interaction	4	20% (0% - 59%)	14% (0% - 47%)	35% (0% - 75%)	31% (0% - 70%)	15% (0% - 70%)	11% (0% - 62%)	53% (0% - 100%)	47% (0% - 100%)
Continuous covariates	4	22% (0% - 60%)	14% (0% - 48%)	35% (0% - 71%)	30% (0% - 65%)	16% (0% - 72%)	11% (0% - 63%)	55% (0% - 100%)	48% (0% - 100%)
Binary covariates	4	11% (0% - 41%)	6% (0% - 30%)	25% (0% - 61%)	23% (0% - 56%)	12% (0% - 64%)	9% (0% - 59%)	47% (0% - 100%)	41% (0% - 100%)
False Positive Rates (FPR)	292	1% (0% - 3%)	1% (0% - 2%)	2% (0% - 3%)	1% (0% - 3%)	3% (0% - 19%)	3% (0% - 19%)	13% (0% - 31%)	11% (0% - 30%)
Covariates correlated with associated covariates	50	1% (0% - 6%)	1% (0% - 4%)	3% (0% - 7%)	2% (0% - 7%)	8% (0% - 54%)	7% (0% - 54%)	29% (0% - 83%)	25% (0% - 80%)
Covariates uncorrelated with associated covariates	242	1% (0% - 3%)	1% (0% - 2%)	1% (0% - 3%)	1% (0% - 3%)	2% (0% - 12%)	2% (0% - 12%)	9% (0% - 20%)	8% (0% - 19%)
Continuous covariates	146	1% (0% - 4%)	1% (0% - 2%)	2% (0% - 4%)	2% (0% - 4%)	4% (0% - 20%)	3% (0% - 20%)	13% (0% - 32%)	11% (0% - 31%)
Binary covariates	146	1% (0% - 3%)	1% (0% - 2%)	2% (0% - 4%)	1% (0% - 3%)	3% (0% - 19%)	3% (0% - 18%)	12% (0% - 30%)	10% (0% - 29%)
